# Research Progress on the Major Histocompatibility Complex in Herbivores: Structure, Genetics, and Disease Resistance

**DOI:** 10.3390/biology15171450

**Published:** 2026-08-24

**Authors:** Manna Dou, Xiangnan Zhou, Junjie Liu, Muhammad Zahoor Khan, Changfa Wang, Xinhao Zhang

**Affiliations:** College of Agriculture and Biology, Liaocheng University, Liaocheng 252000, Chinazahoorkhan@lcu.edu.cn (M.Z.K.)

**Keywords:** major histocompatibility complex, MHC polymorphism, ruminants, non-ruminant herbivores, antigen presentation, disease resistance, molecular breeding, conservation genetics

## Abstract

The major histocompatibility complex (MHC) represents a critical genetic locus that orchestrates adaptive immune responses through antigen presentation and T cell activation, characterized by exceptional polymorphism and species-specific diversity. In herbivores, MHC polymorphisms constitute fundamental determinants of resistance to parasitic, viral, and bacterial infections while serving as crucial indicators of genetic diversity and breeding value in animal populations. Recent advances in high-throughput sequencing technologies, single-cell omics, and population genetics have substantially advanced understanding of MHC gene structure, allelic polymorphism, evolutionary patterns, and associations with disease susceptibility and vaccine responsiveness. This comprehensive review systematically examines the structural characteristics and regulatory mechanisms of MHC genes, their associations with infectious disease resistance, their role in vaccine responses, and their practical applications in molecular breeding programs, conservation genetics, and immunological research. We identify current technical limitations and outline future research directions to establish a robust theoretical foundation for disease resistance breeding and immune regulation strategies in herbivore populations.

## Simple Summary

Herbivores such as cattle, sheep, goats, horses, and donkeys are vital to farming and to many natural ecosystems, yet they are constantly challenged by infections caused by parasites, viruses, and bacteria. Whether an individual animal can fight off a particular infection depends heavily on a family of immune genes, called the major histocompatibility complex, that help the body recognize foreign invaders and trigger the right defensive response. These genes vary widely between individuals: some versions make an animal more resistant to a given disease, while others leave it more vulnerable. In this review, we bring together recent research on how these immune genes are organized, how they are passed from parents to offspring, how they have changed over evolutionary time, and how they shape an animal’s likelihood of becoming ill or responding well to vaccination. We also describe new laboratory tools that are making these complex genes easier to study. This knowledge can help farmers breed healthier, more disease-resistant livestock, help conservationists safeguard endangered species, and guide the design of more effective vaccines. Ultimately, the work supports more sustainable animal agriculture, better animal welfare, and the protection of wildlife.

## 1. Introduction

The major histocompatibility complex (MHC) is present across all jawed vertebrates and constitutes one of the most polymorphic gene regions in the vertebrate genome, fundamentally determining tissue compatibility and orchestrating immune responses [1,2]. MHC molecules execute critical immune functions through antigen recognition, immunosurveillance, and host–pathogen interactions. The historical foundation of MHC research began in 1948 when George Snell demonstrated that transplanted tumors between genetically distinct mouse strains triggered host-mediated rejection, leading to the identification of a specific chromosomal region designated the histocompatibility-2 (H-2) complex, the murine MHC [3]. Subsequently, in 1958, Jean Dausset identified human leukocyte antigen (HLA) system through serological studies of transfused patients and pregnant women, recognizing its corresponding role to the MHC in humans [4]. The seminal structural biology investigations by Don Wiley and Jack Strominger revealed that MHC molecules possess a characteristic peptide-binding groove capable of specifically binding and presenting antigen fragments, providing the molecular foundation for understanding MHC-mediated immune recognition [5].

As the central genetic component of adaptive immunity, the MHC has become a focal point for understanding host–pathogen coevolution, disease susceptibility, and evolutionary adaptation. Among vertebrates, herbivorous mammals occupy a unique ecological and economic position. However, these animals are continuously exposed to diverse parasites and pathogens, which impose strong selective pressures on immune-related genes and threaten both wildlife conservation and livestock productivity. Herbivore MHC evolution is marked by structural conservation, high polymorphism, and multiple evolutionary mechanisms. Its diversity is shaped by pathogen-mediated balancing selection, gene conversion, homologous recombination, copy number variation (CNV), and structural variation (SV), and is further influenced by ecological factors such as habitat and life-history traits [6,7]. These processes have produced species-specific MHC architectures and immune strategies. And MHC genetic polymorphism and its associations with disease susceptibility are often highly species-, breed-, and population-specific, including parasite resistance in ovine populations [8], bovine mastitis susceptibility [9], and viral evasion mechanisms in equine herpesvirus infections [10]. Consequently, systematic investigation of herbivore MHC genetics provides invaluable insights for wildlife conservation and sustainable livestock production. This review synthesizes current knowledge on herbivore MHC structure, function, and genetic variation, integrating recent technological advances to illuminate immune response mechanisms, disease susceptibility, and future applications in disease prevention strategies and selective breeding programs.

## 2. MHC Gene Structure and Function

The MHC region is one of the most polymorphic and gene-dense regions of the vertebrate genome and plays a central role in immune recognition and adaptive immunity [11]. Adaptive immune responses are initiated through antigen presentation, in which MHC molecules display processed peptide antigens for recognition by T-cell receptors, thereby triggering antigen-specific immune responses [12]. Based on their structure and function, MHC genes are classified into Class I, Class II, and Class III regions [13,14]. Among them, MHC Class I and Class II molecules are responsible for peptide antigen presentation to T lymphocytes, whereas MHC Class III genes mainly encode immune-related proteins, including complement components and cytokines, that participate in innate immune responses [15,16,17].

### 2.1. MHC Class I Antigen Presentation Pathway

**Figure 1:**
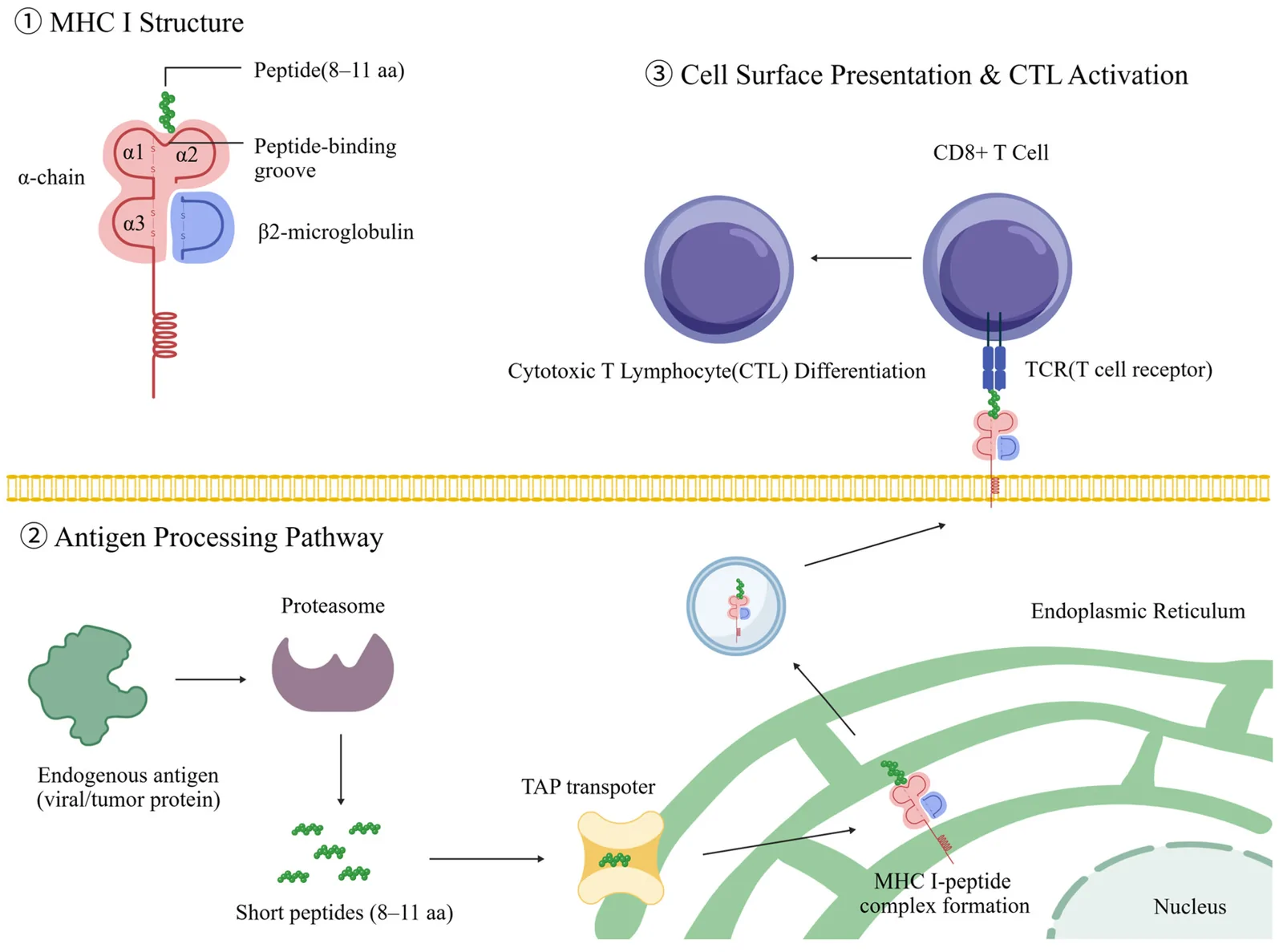
MHC Class I molecular structure and antigen presentation pathway. The schematic illustrates the α-chain domains (α1, α2, α3) and β2-microglobulin structure, the peptide-binding groove, and the intracellular processing pathway from proteasomal degradation through TAP transport to cell surface presentation. The arrows indicate the direction of antigen processing, peptide transport, and MHC I-mediated antigen presentation.

MHC Class I molecules are heterodimers composed of a polymorphic α-chain associated with β2-microglobulin [18,19]. The α1 and α2 domains form a closed peptide-binding groove that preferentially accommodates peptides of 8–11 amino acids [20,21]. MHC Class I molecules primarily present endogenous antigens, including intracellular viral and tumor-derived proteins. Following intracellular processing and peptide loading in the endoplasmic reticulum, peptide–MHC Class I complexes are transported to the cell surface, where they are recognized by CD8^+^ T cells, leading to cytotoxic immune responses (Figure 1) [22,23,24].

### 2.2. MHC Class II Antigen Presentation Pathway

**Figure 2:**
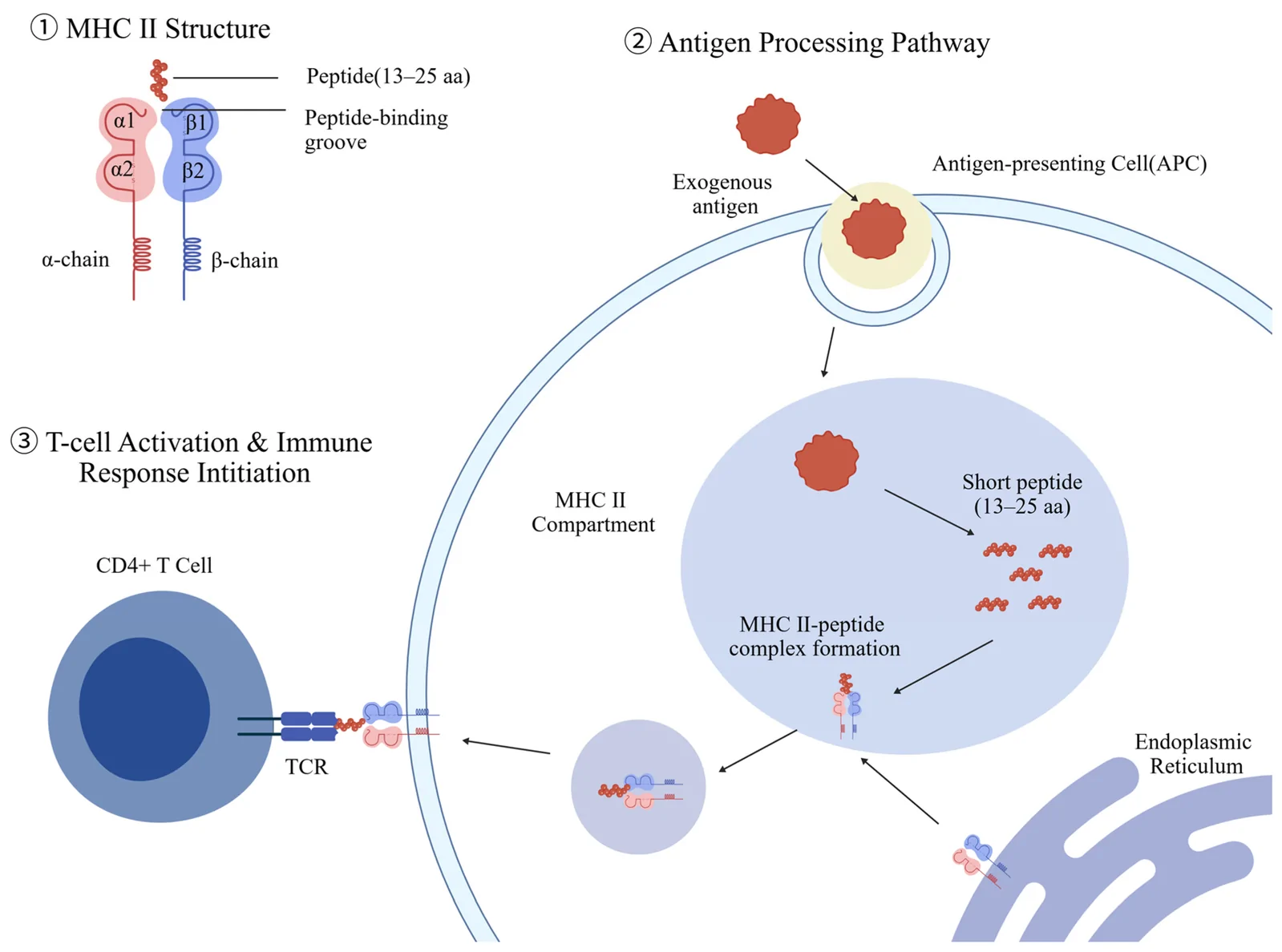
MHC Class II molecular structure and antigen presentation pathway. The schematic shows the α-chain (α1–α2 domains) and β-chain (β1–β2 domains) composition, the open peptide-binding groove structure, and the exogenous antigen processing pathway from endosomal trafficking through peptide binding to cell surface presentation. The arrows indicate the direction of antigen processing, petide transport, and MHC I-mediated antigen presentation.

MHC Class II molecules are heterodimers consisting of α- and β-chains, with the peptide-binding groove formed by the α1 and β1 domains [25]. Compared with MHC Class I, the groove is open at both ends, allowing the binding of longer peptides, typically 13–25 amino acids [26]. MHC Class II molecules primarily present exogenous antigens that have been internalized and processed by professional antigen-presenting cells. Peptide–MHC Class II complexes are subsequently expressed on the cell surface for recognition by CD4^+^ T cells, thereby initiating helper T-cell responses and adaptive immunity (Figure 2) [27,28,29,30,31,32].

### 2.3. MHC Class III Functions

Unlike MHC Class I and Class II molecules, MHC Class III region products do not directly present antigens; rather, they function in innate immune responses through complement cascade components (C2, C4, BF) and tumor necrosis factor superfamily members (TNF, LTB, LTA) [33,34,35]. Additional MHC Class III products, including lymphocyte antigen 6 (LY6) family members [36], LST1 [37], and allograft inflammatory factor 1 (AIF1) [38], participate in immune and inflammatory regulatory processes. Furthermore, six MHC Class III region genes (*DDX39B*, *DXO*, *LSM2*, *NELFE*, *PRRC2A*, SKIV2L) encode RNA-binding proteins that regulate post-transcriptional gene expression and RNA homeostasis [39].

## 3. Genetic Inheritance and Molecular Evolution of the MHC

The MHC region exhibits exceptional genetic polymorphism and gene density within the vertebrate genome, functioning as a central node in immune recognition and response [40]. While MHC inheritance follows classical Mendelian patterns, it demonstrates distinctive characteristics that generate and sustain genetic diversity [41]. MHC loci are typically inherited as haplotypic blocks, with allelic variants on individual chromosomes transmitted together due to extensive linkage disequilibrium [42]. In most species, these haplotypes exhibit codominant expression in heterozygous individuals, enabling presentation of a broader repertoire of antigens [43]. However, the polymorphism and complexity of MHC are not solely caused by simple nucleotide mutations. Its diversity mainly results from the combined influence of pathogens, gene replication, recombination, and natural selection. These processes jointly shape the allelic diversity and genomic structure within vertebrate species [44]. For ease of reference, the types of herbivore MHC are summarized in Table 1.

The molecular evolution of MHC genes is driven by multiple mechanisms operating at both the sequence and genomic levels. At the sequence level, point mutations, homologous recombination, and gene conversion continuously generate novel allelic variation, providing the genetic basis for MHC diversification. Deep sequencing of the human MHC region has shown that its mutation rate is substantially higher than the genomic average, highlighting de novo mutations as an important source of MHC polymorphism [45]. In particular, gene conversion, which occurs predominantly within the peptide-binding regions (PBRs), especially exon 2 of the MHC class II *DRB* gene, can simultaneously replace multiple adjacent codons and rapidly generate new combinations of antigen-binding residues, thereby accelerating the emergence of functionally distinct MHC alleles [46]. At the genomic level, gene duplication, CNV, and SV further drive MHC evolution by reshaping gene content, genomic organization, and haplotype structure. Comparative genomic studies in ungulates have shown that repeated gene duplication and CNV contribute to the expansion of MHC class II genes, particularly *DQA*, *DQB*, and *DRB*, whereas SVs and repetitive elements increase haplotypic complexity and promote lineage-specific diversification of the MHC region [6]. Meanwhile, comparative genomic studies have shown that MHC evolution in ruminants is jointly shaped by ecological and life-history factors, including habitat, diet, and lifespan, with gene duplication and CNV serving as important mechanisms driving its adaptive evolution [7]. The diversity generated by these molecular mechanisms is subsequently maintained by natural selection. Balancing selection, pathogen-mediated selection, and rare-allele advantage collectively preserve the extraordinary polymorphism of MHC genes [47,48]. Over long evolutionary timescales, persistent balancing selection preserves ancient allelic lineages across speciation events, giving rise to trans-species polymorphism (TSP), one of the hallmarks of MHC evolution [49]. Furthermore, the continuous changes in the pathogen community and environmental conditions, especially those related to climate change, constantly generate new selection pressures, driving the continuous adaptive evolution of MHC genes [47].

## 4. Genetic Evolution of MHC in Herbivore Populations

### 4.1. Ruminant MHC

Ruminant species constitute a diverse herbivore subfamily spanning domestic livestock (cattle, sheep, goats) and wild species (deer, antelopes, gazelles), inhabiting heterogeneous ecological niches and confronting varied pathogenic challenges. Among immune-related genomic regions, MHC is one of the most polymorphic loci in vertebrate genomes and plays a central role in adaptive immunity. The unique genomic organization and extensive genetic diversity of ruminant MHC have made it a key model for investigating molecular evolution, population genetic diversity, and host adaptation. Moreover, MHC polymorphisms have been widely associated with disease resistance and susceptibility, highlighting their importance for livestock breeding, disease control, vaccine development, and wildlife conservation [50].

#### 4.1.1. Gene Structure and Evolutionary Characteristics

Ruminant MHC Class II genes exhibit a distinctive genomic organization compared with those of most other mammals. Unlike the contiguous Class II region observed in primates, carnivores, rodents, and many other mammalian species, the ruminant MHC Class II region is divided into two subregions (IIa and IIb) by an ancestral chromosomal inversion [51,52,53,54,55]. A similar genomic organization has also been identified in white-tailed deer, in which the discovery of novel *MHC-DRB* and *MHC-DOB* genes further supports the conservation of this inversion within the family Cervidae [56]. This inversion phenomenon appears to be conserved in cetacean species including finless porpoises, orcas, and seven additional cetacean taxa [57,58], but remains absent in suiform species such as pigs [59], suggesting that MHC Class II inversion occurred subsequent to the porcine-cetacean divergence but prior to cetacean-ruminant speciation events.

**Figure 3:**
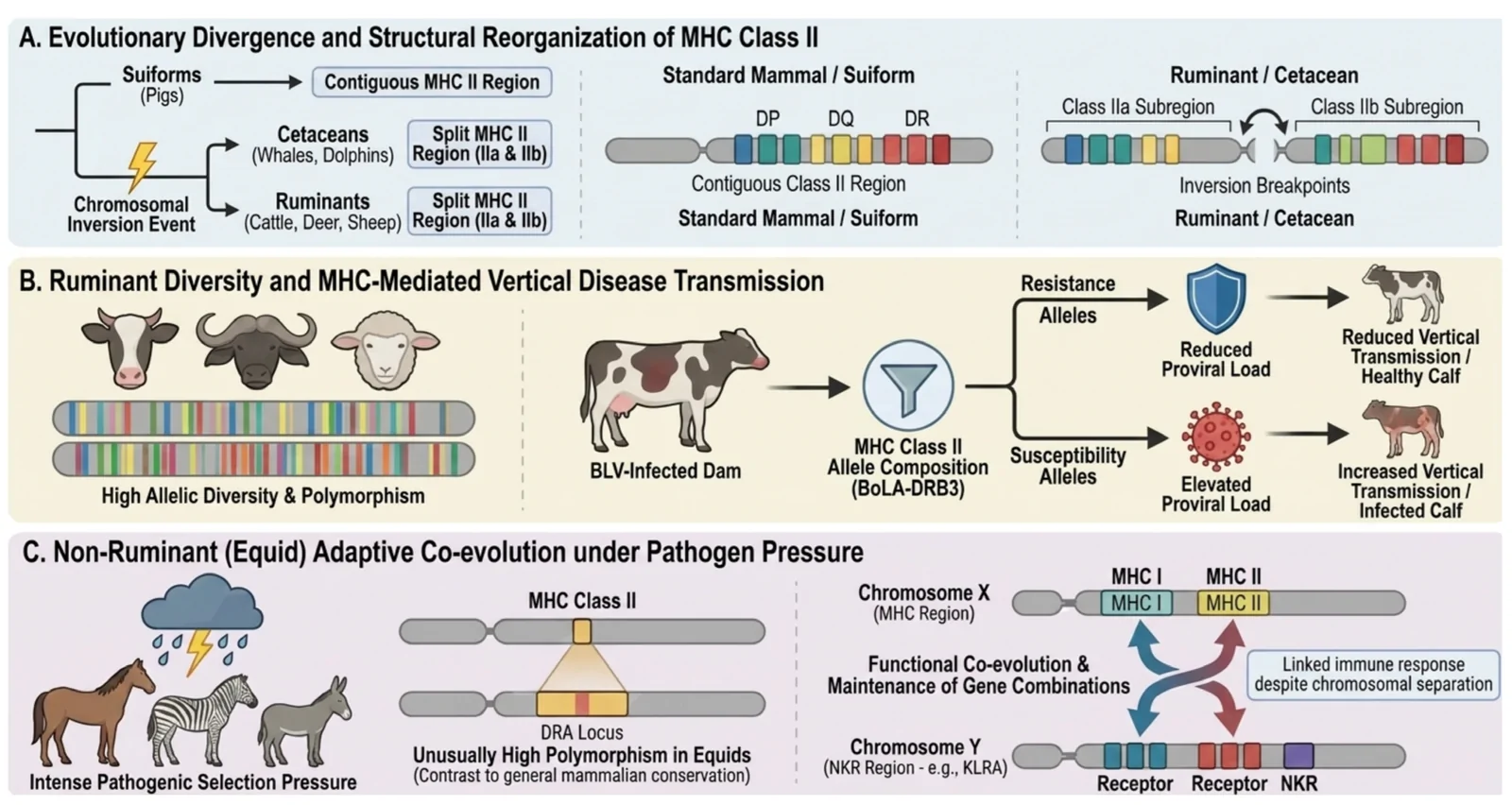
Evolutionary genetics, structural reorganization, and functional implications of the Major Histocompatibility Complex (MHC) in ruminant and non-ruminant herbivore populations. (**A**). Evolutionary divergence and structural reorganization of MHC class II. An ancient chromosomal inversion differentiates ruminants and cetaceans from suiforms (pigs), resulting in a split MHC class II region (IIa and IIb) in ruminants and cetaceans, whereas suiforms retain a contiguous class II organization. (**B**). Ruminant MHC diversity and disease association. Ruminants exhibit high MHC polymorphism. Specific class II alleles (e.g., *BoLA-DRB3* in cattle) are associated with differential outcomes of Bovine Leukemia Virus (BLV) infection, affecting proviral load and vertical transmission risk. (**C**). Non-ruminant adaptive immune evolution under pathogen pressure. Equids show strong pathogen-driven selection, including elevated polymorphism at the MHC class II *DRA* locus and coordinated evolution between MHC and Natural Killer Receptor (NKR) gene systems. Overall, these patterns reflect lineage-specific immunogenetic divergence associated with structural variation in the MHC region.

Chromosomal inversions are recognized as important drivers of genome evolution because they suppress recombination, preserve co-adapted gene complexes, and facilitate lineage divergence [60,61]. Accordingly, the MHC Class II inversion in ruminants has been proposed to contribute to the long-term structural evolution of the MHC region and to promote independent evolutionary trajectories in ruminant and cetacean lineages [62] (Figure 3). However, the functional consequences of this genomic rearrangement, particularly its effects on MHC diversity, gene regulation, and disease resistance, remain incompletely understood and warrant further investigation.

#### 4.1.2. Genetic Diversity and Evolutionary Patterns

Bovine leukocyte antigen (BoLA) research has concentrated on Holstein-Frisian cattle. Analyses of the highly polymorphic *BoLA-DRB3* locus have demonstrated clear genetic differentiation among Holstein-Friesian populations adapted to subtropical and temperate environments, indicating that environmental conditions and pathogen pressures have contributed to population-specific MHC evolution [63]. Similarly, integrated analyses of MHC Class I and Class II haplotypes in Zambian indigenous cattle identified 258 complete MHC haplotypes, including 170 previously unreported haplotypes, highlighting the extraordinary genetic diversity maintained in indigenous populations [64]. This extensive haplotypic variation is thought to result from long-term balancing selection acting under heterogeneous pathogen environments together with relatively weak artificial selection, thereby preserving a broad antigen-presenting repertoire and enhancing population adaptability to diverse infectious challenges [65].

Comparable evolutionary patterns have also been observed in other ruminants. In buffalo, characterization of the DQA region identified three distinct loci (*Bubu-DQA1*, *DQA2*, and *DQA3*), while phylogenetic analyses suggested convergent evolution of bovine and buffalo MHC alleles together with similar patterns of copy number variation and allelic polymorphism [66]. Likewise, ovine *DRA* genes exhibit trans-species polymorphism [67], whereas caprine *DQB1* exon 2 has evolved under strong balancing selection, and previously undetected *DRB1* alleles have been identified in Iberian goat populations, indicating the persistence of cryptic genetic diversity despite historical population bottlenecks [68,69]. Collectively, these findings demonstrate that balancing selection, structural variation, and historical demographic processes have jointly shaped the remarkable MHC diversity observed across ruminant species.

The extensive MHC diversity generated through long-term evolution has important functional consequences for host immunity. Differences among MHC haplotypes determine the repertoire of peptides that can be presented to T cells, thereby influencing immune recognition of viral, bacterial, and parasitic pathogens [70]. Consequently, numerous studies have investigated associations between specific MHC alleles or haplotypes and disease susceptibility or resistance in ruminants. Once robust genotype–phenotype relationships have been established and functionally validated, favorable MHC variants can be incorporated into marker-assisted selection or genomic selection programs to simultaneously improve disease resistance and production performance, providing a foundation for precision breeding in livestock.

### 4.2. Non-Ruminant Herbivore MHC

The research on the genetic evolution of MHC in non-ruminant herbivores mainly focuses on equine species. However, the research on non-ruminant herbivores other than equines is relatively limited, but it still reveals some common patterns of MHC evolution among different herbivorous animals. Equidae family members, including horses, donkeys, and zebras, represent prototypical non-ruminant herbivores whose MHC genetics, designated equine leukocyte antigen (ELA), exhibit pronounced polymorphism and interspecific allele sharing among MHC Class II genes (*DRA* and *DQA*) [71]. Pathogen-mediated selection pressures generate exceptionally high MHC gene diversity in equine populations [48], providing empirical support for pathogen-driven MHC evolution based on population genetic diversity analysis and documented pathogen-MHC associations. These characteristics establish equids as exemplary model organisms for investigating immune genome MHC diversity and evolution [48,71]. The MHC gene cluster of rabbits has a relatively compact structure. The *DRB* gene is the main functional gene in the Class II region. Its polymorphism is mainly driven by point mutations and positive selection, and it exhibits typical cross-species polymorphism, indicating that some MHC allelic lineages have already formed before species differentiation and have been preserved under the long-term balancing selection [72]. The MHC genetic evolution patterns of non-ruminant herbivores (including horses, rabbits, etc.) not only reflect the adaptive responses of the host immune system under different pathogen environments, but also demonstrate the combined influence of population history, genetic drift, and life history characteristics on MHC genetic variation [73,74].

#### 4.2.1. Adaptive Genetic Evolution Under Pathogenic Selection Pressure

The immune genome, encompassing genes involved in innate and adaptive immunity, evolves under strong pathogen-mediated selective pressure. Equids, including domesticated horses, free-ranging populations, and captive species, have experienced diverse environmental conditions and pathogen exposures throughout their evolutionary history, making them valuable models for investigating immune gene evolution [75,76]. Comparative analyses of equine MHC Class II genes have revealed distinct evolutionary patterns among different loci. While the relatively conserved *DRA* gene exhibits unexpectedly high allelic polymorphism, the *DQA* gene shows strong signatures of positive selection concentrated within antigen-binding sites, reflecting adaptive diversification driven by pathogen-mediated selection [71]. In addition, significant trans-species polymorphism has been identified among horses, zebras, and donkeys [71], indicating that several MHC allele lineages originated before species divergence and have been maintained through long-term balancing selection. Together, positive selection and trans-species polymorphism have promoted the functional diversification of antigen-binding regions while preserving allelic lineages that enhance immune recognition of diverse pathogens.

Beyond the evolutionary diversification of MHC genes, increasing evidence suggests functional interactions between MHC and natural killer cell receptor (NKR) gene families. Although MHC and NKR genes are located on different chromosomes, they are thought to co-evolve under long-term pathogen-mediated selection because of their complementary roles in regulating innate and adaptive immune responses [73,77]. The killer cell lectin-like receptor subfamily A (KLRA) genes encode Ly49 family receptors expressed on natural killer cells that recognize MHC class I molecules [78]. Population genetic analyses of Camargue, African, and Romanian horse populations identified significant associations between MHC and NKR loci, confirming KLRA3 as an important functional ligand of equine MHC [79]. These findings indicate that coordinated evolution between MHC and NKR genes has contributed to immune adaptation in equids and highlight the importance of considering epistatic interactions among immune gene families when investigating disease resistance.

#### 4.2.2. Genetic Variation and Polymorphic Characteristics

The equine MHC Class II DRA gene is generally characterized by limited sequence variation across most mammalian species but exhibits unexpectedly high levels of polymorphism in equids, suggesting lineage-specific evolutionary diversification [80]. Population-based analyses of horses, donkeys, and zebras have further revealed substantial genetic diversity at MHC Class II loci, including *DRA*, *DRB*, and *DQB*, indicating that pathogen-mediated selection has shaped the evolutionary trajectory of equine MHC genes [81,82,83,84]. Moreover, resequencing analyses integrating previously published genomic resources identified signatures of positive selection at putatively functional *DRB* and *DQB* loci, while simultaneously improving annotation of the equine MHC region. These findings provide important insights into the molecular mechanisms underlying MHC evolution and adaptive immune diversification in equids [85,86].

Beyond elucidating evolutionary patterns, several studies have investigated the functional significance of equine MHC diversity. A population genetic analysis of donkeys exposed to *Babesia caballi* infection characterized the allelic diversity of the *MHC-DRA* locus and demonstrated extensive variation within equine *DRA* genes, providing a valuable resource for exploring host–pathogen interactions [81]. In addition, transcriptomic analysis of lymphocytes from ELA-homozygous horses identified polymorphisms in MHC Class II *DQ* and *DR* genes among five major ELA haplotypes while showing that the overall complement of *DQ* and *DR* genes remains highly conserved across haplotypes [87]. These studies establish a foundation for investigating the relationships between MHC polymorphism, immune function, and disease susceptibility in equids.

## 5. MHC-Disease Associations in Herbivores

Parasitic infections constitute serious threats to livestock health and animal husbandry sustainability, with parasite resistance in ovine populations generating substantial research interest [88]. Analyses of associations between ovine MHC haplotypes and fecal egg count revealed that mutations within DRB1*1101 and DQB2*GU191460 alleles or flanking genomic regions confer nematode infection resistance, with six antigen-binding amino acid substitutions in DRB1*1101 demonstrating significant associations with reduced adult *Teladorsagia circumcincta* burdens [89].

Contagious ovine digital dermatitis (hoof rot) affects ungulate hoof integrity with severity ranging from mild inflammation to severe lameness and feeding inability [90]. Comprehensive analysis of *MHC-DQA2* variations in conjunction with hoof rot status and infection severity across ovine breeds identified that *MHC-DQA2* allele *1101 associates with elevated hoof rot susceptibility, whereas allele *1201 correlates with reduced susceptibility [91]. These findings demonstrate that MHC genetics substantially influences ovine hoof rot susceptibility with breed-dependent variation, suggesting that selective breeding utilizing *MHC-DQA2* allele or haplotype information can reduce ovine digital disease incidence [91].

Bovine mastitis, predominantly caused by bacterial pathogens, represents a disease substantially compromising animal health, encompassing clinical manifestations from severe clinical mastitis to subclinical infections, with subclinical mastitis accounting for 70–80% of mastitis-related economic losses [92]. Consistent with the distinct immunopathological features of subclinical mastitis, MHC class II-positive neutrophils preferentially accumulate in milk during subclinical rather than clinical mastitis and show a strong positive correlation with T lymphocyte infiltration, suggesting an important role for MHC-mediated antigen presentation in local adaptive immune responses [93]. Accordingly, the highly polymorphic *BoLA-DRB3* gene, a key MHC class II locus involved in antigen presentation, has been extensively investigated for its association with disease resistance, particularly susceptibility to mastitis in cattle and buffalo [94]. However, although numerous studies have linked *BoLA-DRB3* alleles to disease resistance, the reported associations are often inconsistent across breeds, populations, pathogens, and disease phenotypes [41,95]. These discrepancies indicate that MHC–disease associations are context-dependent rather than universally applicable, highlighting the need for population-specific validation before MHC markers can be reliably incorporated into livestock genetic research and breeding programs. Beyond *BoLA-DRB3*, advances in targeted next-generation sequencing have expanded the search for mastitis-associated loci within the bovine MHC region. Resequencing studies have identified five candidate genes (*POU5F1*, *IER3*, *GNL1*, *ABCF1*, and *PRR3*) associated with mastitis susceptibility, all located within the BoLA class I region [96]. Among these, *POU5F1* and *IER3* have been supported by both association analyses and preliminary functional evidence, making them promising candidates for improving mastitis resistance. In contrast, *ABCF1*, *GNL1*, and *PRR3* have primarily been identified through GWAS and gene network analyses, and their causal roles remain to be established through functional validation, including gene expression studies, genome editing, and animal models [96].

Systematic examination of specific contradictory findings reveals several recurring patterns underlying reported discrepancies in MHC-disease associations. Regarding *BoLA-DRB3* and mastitis resistance, certain alleles (e.g., DRB3*011:01) have been linked to protection in some Central European Holstein populations but to elevated susceptibility in Brazilian Girolando cattle; this contradiction likely reflects differences in predominant mastitis pathogens (coagulase-negative staphylococci versus Streptococcus agalactiae) and regional microbiome composition [41,95]. In ovine nematode resistance, *OLA-DRB1* alleles correlated with reduced *Teladorsagia circumcincta* burdens in Scottish Blackface sheep showed no protective effect in Merino breeds exposed to the same parasite species, suggesting that breed-specific MHC peptide-binding groove architecture differentially accommodates parasite-derived antigens [89]. For equine insect bite hypersensitivity, ELA-II risk haplotypes consistently associated with disease in Icelandic and Dutch Warmblood horses were not confirmed in Friesian horses, possibly because geographic variation in Culicoides midge allergen repertoire alters the antigenic landscape encountered by MHC molecules [97]. Pathogen strain variation also contributes substantially: *BoLA-DRB3* associations with foot-and-mouth disease susceptibility differ across FMD serotypes O, A, and Asia-1, indicating that allele-specific antigen presentation efficiency is antigen-dependent and cannot be generalized across viral variants [98,99,100]. Methodological heterogeneity further compounds cross-study comparability: PCR-RFLP and serological typing methods used in earlier studies frequently fail to resolve alleles that modern high-resolution NGS distinguishes unambiguously, artificially inflating apparent inconsistencies between historical and contemporary datasets. Finally, inconsistent disease phenotype definitions (e.g., clinical versus subclinical mastitis; parasite egg count versus clinical disease severity) and limited sample sizes in many original studies constrain statistical power and reproducibility. A representative summary of conflicting MHC-disease association findings and their probable explanatory factors is provided in Table 2.

Foot-and-mouth disease (FMD) constitutes an economically devastating, highly contagious condition affecting cloven-hoofed animals including domesticated cattle, sheep, and goats [101]. The BoLA system mediates critical antigen presentation functions and orchestrates immune responses against FMD [102]. Extensive investigations demonstrate that *BoLA DRB3* Class II antigen-encoding allele polymorphisms associate with FMD resistance or susceptibility [98,99]. Additional studies identified five *BoLA DRB3* exon 2 genotypes as candidate genetic markers for FMD resistance determination, discovering that *BoLA-DRB3* genotype HaeIII AA provides relative protective effects against buffalo FMD virus serotype O infection [100].

In cattle, bovine leukemia virus (BLV) infection is strongly associated with polymorphisms in the MHC Class II *BoLA-DRB3* gene. Studies in Holstein cattle demonstrated that *BoLA-DRB3* variation shows a stronger association with BLV proviral load than polymorphism at the *DQA1* locus, indicating that *DRB3* is a major immunogenetic determinant of host response to BLV infection [103]. Specific *BoLA-DRB3* alleles have been identified as resistance- or susceptibility-associated variants, with resistance alleles correlating with lower proviral loads and susceptibility alleles correlating with higher viral burdens [103,104]. These findings suggest that naturally occurring MHC polymorphisms influence BLV infection dynamics and disease susceptibility, highlighting the potential value of *BoLA-DRB3* as a genetic marker for BLV control and disease-resistance breeding in cattle [103,104].

Equine herpesvirus type 1 (EHV-1), an alpha-herpesvirus within the Herpesviridae family, causes respiratory disease, abortion, and encephalomyelitis in horses [105]. Equine MHC Class I molecules function as EHV-1 entry receptors, executing critical roles in viral cell entry. Studies demonstrated that equine MHC Class I molecules directly bind EHV-1 glycoprotein D (gD), with MHC Class I surface expression inhibition substantially reducing viral infection rates [105]. Furthermore, amino acid position 173 within the MHC Class I α2 domain constitutes a determinant of EHV-1 cell entry efficiency [106]. The EHV-1-encoded alpha-herpesvirus protein pUL56, derived from ORF1, represents a novel immune escape mechanism that actively downregulates cell surface MHC Class I through novel endocytic pathways, thereby evading host immune surveillance—a process blocked by inhibiting ubiquitin-activating enzyme E1 necessary for ubiquitination [107]. These investigations provide mechanistic insights into EHV-1 immune evasion and identify potential antiviral therapeutic targets [107].

Equine sarcoidosis constitutes one of the most prevalent cutaneous neoplastic conditions in equids, characterized as a non-metastatic persistent fibroblast tumor exhibiting high recurrence rates and treatment difficulty [108,109]. Comprehensive genotyping and association analyses of microsatellite loci and two MHC Class II genes (*DRA*, *DQA1*) within MHC Classes I, II, III subregions, alongside natural killer cell receptor genes (KLRA, CLEC subregions), demonstrated that MHC Class I, Class II, and KLRA molecules contribute to innate immune responses in equine sarcoidosis [110]. Numerous studies link equine MHC Class II regions to additional important conditions including uveitis [111], insect bite hypersensitivity [97], and nodular skin disease [112]. These observations advance understanding of equine MHC Class II region-disease associations [97,110,111,112].

MHC genetics substantially influences alloimmune responses following equine transplantation; studies demonstrated that allogeneic mesenchymal stem cells (MSCs) cross-react with non-donor MHC types in vivo, potentially limiting allogeneic MSC therapeutic efficacy and generating adverse inflammatory responses in recipients [113,114,115]. MSCs represent ideal cellular sources for treating equine musculoskeletal injuries [116], with therapeutic properties derived from immunomodulatory and nutritional factors secreted by cells [117,118,119]. To circumvent MHC-mismatch immune rejection of allogeneic MSCs and improve therapeutic efficacy and safety, investigations demonstrated that transforming growth factor-beta 2 (TGF-β2) treatment downregulates MSC surface MHC expression, potentially promoting allogeneic therapy and establishing novel therapeutic approaches for equine musculoskeletal injury treatment [120]. Table 3 provides a comparative mechanistic analysis of how MHC allelic variants generate differential disease resistance and susceptibility phenotypes across herbivore species, synthesizing molecular mechanisms, immune outcomes, and clinical consequences documented across parasitic, bacterial, viral, and neoplastic disease challenges.

## 6. Future Research Directions and Practical Applications

Recent technological advances in high-throughput sequencing, single-cell omics, and population genetic analysis have substantially elevated MHC research capacity, rendering herbivore MHC’s critical role in immune responses and disease resistance increasingly prominent. Future investigations will prioritize high-throughput sequencing approaches to comprehensively analyze complete herbivore MHC genome structure and facilitate its application in molecular breeding, conservation genetics, and immunological research. Meanwhile, artificial intelligence technology is gradually becoming an important tool for the study of MHC functions. In recent years, deep learning models such as NetMHCpan have significantly improved the prediction accuracy of MHC–peptide binding affinity and antigen presentation ability [121]. Furthermore, protein structure prediction algorithms such as AlphaFold can accurately analyze the three-dimensional structure of MHC molecules, providing a new research approach for elucidating the conformational changes in the binding grooves of different alleles and their antigen-binding specificity [122]. In the future, emerging technologies such as artificial intelligence are expected to further integrate multi-source data including genetic variations, protein structures, immune peptide groups and immune phenotypes, enabling precise prediction of MHC functions and the elucidation of molecular mechanisms.

### 6.1. Molecular Breeding Applications

In future animal breeding programs, once disease-associated MHC alleles have been comprehensively identified and functionally validated, marker-assisted selection (MAS) could serve as an important tool for incorporating favorable MHC variants into breeding schemes to improve disease resistance. For example, the *BoLA-DRB3* allele has been associated with resistance or susceptibility to multiple infectious diseases, including mastitis, trypanosomiasis, and tick infestation, as well as with economically important production traits in cattle [123]. These findings highlight the potential of *BoLA-DRB3* as a promising selection target for future MHC-based breeding strategies, although further validation across diverse populations and production systems is still required before routine implementation. However, the extreme polymorphism and extensive structural variation in the MHC region represent a double-edged sword for herbivore breeding [124]. While these characteristics underpin adaptive immune diversity, they also complicate accurate genotyping, haplotype reconstruction, causal variant identification, and the development of robust marker-assisted selection strategies. Consequently, future MHC-based breeding is likely to rely on high-resolution long-read sequencing, functional genomics, and genomic selection rather than on single-marker approaches [125,126,127].

The practical implementation of MHC-based breeding programs additionally requires careful consideration of regulatory frameworks and of how MHC information is integrated into existing genetic evaluation schemes. Implementation is contingent on the availability of sufficiently large, functionally validated reference datasets and on the development of multi-trait selection indices that balance disease resistance against production and reproduction performance, avoiding inadvertent negative selection responses. A formal evaluation of the economic implications of MHC-informed selection lies beyond the scope of the present review, which is centered on the structural, evolutionary, and immunological biology of the herbivore MHC, and is identified as a priority for future work in the Conclusion. From a regulatory perspective, MHC genotyping for breeding decisions in most major livestock-producing jurisdictions falls within existing animal identification and breed improvement frameworks and does not typically require independent regulatory approval, provided that genotype data are integrated within a comprehensive zootechnical assessment rather than applied as a standalone selection criterion. However, if MHC-based selection schemes are accompanied by claims of enhanced food safety, improved animal welfare outcomes, or disease-free certification, the labelling, traceability, and certification requirements of national veterinary and agricultural authorities (e.g., EU Regulation 2016/429 on transmissible animal diseases; USDA APHIS animal health improvement frameworks) may become applicable. Breed societies and national genetics evaluation centers should therefore be engaged early in program design to ensure that MHC-based selection targets are compatible with existing genetic evaluation frameworks and do not inadvertently narrow the broader genetic diversity of breeding populations.

### 6.2. Conservation Genetics Applications

In future conservation breeding programs, MHC genotyping could become an important tool for guiding marker-assisted selection (MAS) and genetic management aimed at preserving adaptive genetic variation in endangered herbivores. For example, the endangered Przewalski’s horse exhibits extremely low genetic diversity at the MHC *DQA* locus, with reduced MHC diversity being associated with increased susceptibility to infectious diseases [128]. As high-resolution MHC characterization continues to improve, integrating MHC-based MAS into conservation breeding and reintroduction programs may help maximize adaptive genetic diversity, optimize mating strategies, and enhance the long-term resilience of restored populations.

### 6.3. Immunological and Vaccine Development Applications

MHC executes fundamental roles in herbivore immune responses, particularly in vaccine development and optimization. Bovine neonatal pancytopenia (BNP) represents a serious disease affecting newborn calf health; investigations confirmed MHC Class I presence in vaccines through Western blot analyses using MHC Class I-specific monoclonal antibodies, with MHC Class I peptides (including BVDV antigens) in vaccines capable of triggering BNP-related alloimmunization [129]. These findings demonstrate MHC’s critical roles in vaccine-associated pathology and immunization strategy optimization [129].

### 6.4. Advanced Technological Approaches

To address limited affinity between T cell receptors (TCRs) and peptide-MHC (pMHC) complexes, high-throughput pMHC polymer technology has emerged with extensive applications in disease treatment, vaccine development, and cancer immunology, while enabling comprehensive T cell-mediated immune response monitoring [130]. This technology circumvents cumbersome traditional methodologies through ultraviolet-mediated peptide exchange technology, enabling large-scale parallel pMHC preparation and facilitating successful detection of antigen-specific CD^8+^ T cells in murine antigen-specific splenocyte samples [131]. To further enhance pMHC complex stability, methods based on sortase-mediated enzymatic conjugation and click chemistry have been developed, expanding research tools for antigen-specific T cell recognition and therapeutic targeting, thereby deepening tumor-specific immune response understanding and establishing novel personalized immunotherapy pathways [132]. Novel antigen discovery technology termed T cell receptor antigen mapping (TCR-MAP) enables high-throughput, high-sensitivity capture of MHC Class I-restricted and Class II-restricted TCR responses to self-antigens and pathogenic challenges, substantially accelerating T cell discovery in cancer, infectious disease, and autoimmune contexts [133].

Regarding technical implementation, the complex genomic architecture of MHC genes, characterized by extensive polymorphism, duplicated loci, CNV, SV, and numerous pseudogenes, poses substantial challenges for accurate MHC genotyping in herbivorous animals [7,65,125]. Conventional PCR-based and short-read sequencing approaches are particularly susceptible to allele dropout, amplification bias, paralog misassignment, and pseudogene interference, which can lead to inaccurate allele calling, incomplete haplotype reconstruction, and underestimation of MHC diversity [125,134,135]. These challenges are further exacerbated by the high sequence similarity among duplicated MHC loci and the difficulty of distinguishing functional genes from non-functional copies [125,136]. High-throughput MHC typing technologies have substantially improved the characterization of MHC diversity at the genomic scale [137,138]. In particular, third-generation long-read sequencing (LRS) enables the resolution of highly repetitive MHC regions, facilitating accurate haplotype phasing, structural variation detection, and complete MHC locus assembly while reducing errors caused by paralog ambiguity and allele dropout [139,140]. Meanwhile, next-generation sequencing (NGS) provides high-throughput and cost-effective genotyping and has been successfully applied to high-resolution HLA typing through the integration of whole-genome sequencing and transcriptomic data [141]. Nevertheless, NGS remains limited in resolving highly repetitive regions and complex haplotypes because of its short-read length, whereas LRS is constrained by higher costs, sequencing accuracy, DNA quality requirements, and computational complexity. Therefore, integrating NGS and LRS technologies, together with optimized bioinformatic pipelines, is expected to provide the most robust framework for accurately resolving the complete genomic architecture, haplotypes, and genetic diversity of MHC regions in herbivorous animals [141]. Furthermore, immunoinformatics technology substantially enhances MHC Class I epitope prediction accuracy and efficiency through artificial intelligence integration, multi-omics data synthesis, and advanced algorithmic approaches (NetMHC, IEDB, MHCflurry), accelerating neoantigenic epitope identification, improving immunotherapy accuracy, and enabling personalized vaccine development [142]. MHC-integrated prediction tools progressively enhance cancer vaccine neoantigenic identification, with AI-driven MHC epitope prediction and personalized vaccine development constituting emerging research frontiers in this field [142].

To assist researchers in selecting appropriate genotyping strategies for specific research contexts, Table 4 provides a comparative overview of the sequencing platforms most commonly applied to herbivore MHC typing, summarizing their technical characteristics, analytical strengths, and limitations. For large-scale population-level studies focused on well-characterized loci such as *BoLA-DRB3* exon 2 or *OLA-DRB1*, targeted amplicon sequencing on Illumina platforms (MiSeq or NextSeq) remains the most practical approach, delivering high-throughput, allele-level resolution supported by established and extensively validated bioinformatic pipelines. When comprehensive haplotype phasing, structural variant detection, or de novo characterization of poorly annotated MHC regions is required, third-generation long-read sequencing (PacBio HiFi or Oxford Nanopore) is preferable, as these platforms traverse repetitive MHC architecture and provide full-haplotype resolution without dependence on reference assemblies. Whole-genome short-read sequencing with Illumina instruments provides the broadest genomic coverage and allows MHC genotyping to be extracted from datasets collected for other purposes, but requires high sequencing depth and sophisticated bioinformatic pipelines to resolve MHC complexity reliably. For conservation genetics and wildlife studies involving degraded or low-concentration DNA, capture-based enrichment combined with NGS offers a pragmatic balance of sensitivity and analytical tractability. Platform selection should therefore be guided primarily by the biological question, the genomic complexity of the target species and MHC region, the quality and quantity of available DNA, and the bioinformatic infrastructure accessible to the research group.

### 6.5. Emerging Technologies: CRISPR-Based MHC Editing and Synthetic Biology Approaches

Beyond the characterization of naturally occurring MHC variation, emerging biotechnological approaches offer conceptually novel possibilities for directly modifying or engineering MHC function, and their potential relevance to herbivore immunogenetics warrants consideration alongside appropriate scientific and ethical caution. Although these technologies remain largely experimental and distant from routine livestock application, understanding their scope and current limitations is valuable for positioning the future trajectory of MHC research in the context of broader developments in animal biotechnology.

CRISPR-Cas9 genome editing has emerged as the most precise and accessible tool for targeted modification of genomic loci, including the highly polymorphic MHC region. A foundational proof-of-concept study by Kelton et al. demonstrated that CRISPR-Cas9-induced double-stranded breaks flanking the native murine MHC-I H2-Kd locus enabled scarless exchange of an orthogonal H2-Kb allele in RAW264.7 macrophage cell lines, with the reprogrammed cells retaining full functional capacity for antigen presentation and cognate T cell activation [143]. This study established that CRISPR-Cas9 can be used not merely to knock out MHC loci but to perform allele-level reprogramming—a conceptually important distinction for applications in which the goal is not gene disruption but replacement of a susceptibility-associated allele with a resistance-associated variant. In principle, extending this approach to herbivore MHC systems could offer a route to introducing favorable immune alleles into susceptible populations in which such variants are absent or rare, bypassing the generational timescales required for conventional selective breeding to shift allele frequencies substantially.

The feasibility of precision gene editing in cattle for immune-related traits has been further advanced by broader work integrating immunogenomics with CRISPR/Cas9 technology. Islam et al. reviewed how the systematic identification of resistance-associated immune gene markers through immunogenomic approaches, including MHC association studies, can provide actionable targets for subsequent CRISPR-based functional modification, arguing that the technical accessibility and declining cost of CRISPR-Cas9 enhance its potential as a complement to conventional marker-assisted selection in livestock disease resistance breeding programs [144]. More recently, Workman et al. produced the first live gene-edited calf with dramatically reduced susceptibility to bovine viral diarrhea virus (BVDV) through CRISPR-mediated homology-directed repair introducing a six amino acid substitution in the BVDV-binding domain of bovine CD46, with no detected off-target edits and a normal healthy phenotype at 20 months of age [145]. Although CD46 is not an MHC gene, this landmark study provides the most advanced and rigorously characterized proof-of-concept to date for precision CRISPR editing of a bovine immune receptor gene to confer viral disease resistance in a live animal, illustrating both the biological feasibility and the translational pathway for analogous MHC-focused approaches. Taken together, these studies suggest that CRISPR-mediated introduction or correction of specific MHC alleles in herbivore cell lines and, eventually, embryos is a scientifically tractable objective. However, off-target genomic effects, mosaicism in edited embryos, incomplete editing efficiency, and uncertainty about the long-term immunological consequences of MHC allele substitution in a living animal all remain substantial challenges that must be resolved before any translational application is contemplated.

Synthetic biology approaches represent a more speculative but conceptually significant frontier for MHC research. Rational computational redesign of MHC peptide-binding grooves, using structure-based tools such as Rosetta, ProteinMPNN, and AlphaFold-coupled design pipelines, could in principle generate molecules with enhanced antigen-presenting capacity against specific veterinary pathogens of interest. Papadaki et al. demonstrated a fixed-backbone computational design strategy for engineering chimeric MHC-I molecules that combine peptide-binding and T cell receptor recognition surfaces from distinct existing HLA allotypes, with X-ray crystallography confirming that the resulting chimeric molecules can bind selected peptide antigens in a defined backbone conformation [146]. This work establishes that targeted synthetic modification of the MHC peptide-binding groove can produce structurally validated, functionally coherent molecules, providing a conceptual foundation for applying similar design logic to non-human MHC systems. However, analogous experimental efforts in ruminant or equine MHC systems are entirely lacking, and whether synthetic MHC variants designed against specific veterinary pathogen antigens would perform predictably in the complex immunological context of a living herbivore remains deeply uncertain.

Several critical caveats must accompany any discussion of these emerging approaches. First, the regulatory landscape governing CRISPR-edited livestock is highly restrictive in most major jurisdictions. As comprehensively reviewed by Wray-Cahen et al., the European Union currently applies its genetically modified organism (GMO) regulatory framework to genome-edited animals irrespective of whether foreign DNA is introduced, while the United States FDA evaluates intentional genomic alterations in animals on a case-by-case basis under New Animal Drug provisions—pathways that are lengthy, costly, and carry substantial uncertainty for developers [147]. Although newer regulatory approaches pioneered in Argentina and adopted by a growing number of countries now permit genome-edited organisms that could have arisen through conventional breeding to be regulated as conventional organisms, these frameworks have so far been applied primarily to plants, and their extension to gene-edited livestock remains limited and jurisdiction-specific [147]. Second, the immunological consequences of artificially modifying MHC diversity are unpredictable in ways that extend well beyond the pathogen-resistance phenotype targeted by the original design: altering antigen presentation at one MHC locus may impair recognition of non-target pathogens, disrupt thymic T cell repertoire shaping, increase susceptibility to autoimmune pathologies, or interfere with the MHC-dependent maternal–fetal immune interactions that influence reproductive success in livestock. Third, the introduction of artificially engineered MHC variants into breeding populations raises ethical concerns regarding genetic diversity management and the potential for irreversible population-level consequences that the scientific and regulatory communities have not yet systematically addressed.

In summary, CRISPR-based MHC editing and synthetic biology approaches to MHC engineering represent intellectually compelling research directions that could, in the longer term, expand the practical toolkit for MHC-based disease resistance improvement in herbivores. For the foreseeable future, however, these technologies are best regarded as experimental tools for the functional validation of specific allele-phenotype hypotheses under controlled laboratory conditions rather than near-term applied breeding strategies. Any consideration of translational development must be grounded in rigorous pre-clinical safety evaluation, transparent engagement with diverse stakeholders—including farmers, veterinarians, consumers, and regulators—and careful alignment with the evolving international biosafety and biotechnology governance frameworks that will ultimately determine whether and how these tools can be responsibly deployed in food-producing and wildlife populations.

## 7. Conclusions

This review summarizes current knowledge of MHC in herbivores, with emphasis on its genomic organization, genetic diversity, molecular evolution, and immunological significance. Extensive evidence has established that the MHC is one of the most polymorphic genomic regions in vertebrates and plays an essential role in antigen presentation and adaptive immunity through structurally distinct Class I and Class II pathways. Comparative studies further indicate that evolutionary processes, including gene duplication, chromosomal rearrangements, recombination, gene conversion, and balancing selection, have contributed to the remarkable diversity of MHC genes observed across herbivore species.

Accumulating evidence suggests that MHC polymorphisms are associated with variation in susceptibility or resistance to a range of viral, bacterial, and parasitic diseases. However, many reported MHC–disease associations remain population-, breed-, or species-specific, and relatively few candidate alleles or haplotypes have been functionally validated. Consequently, caution is warranted when extrapolating association studies to broader biological mechanisms or practical breeding applications. Likewise, although advances in high-throughput sequencing, long-read sequencing, and comparative genomics have substantially improved the characterization of complex MHC regions, important knowledge gaps remain regarding structural variation, epigenetic regulation, functional validation of MHC variants, and the evolutionary significance of complex haplotypes.

A further limitation of the present review is that it does not address the economic dimensions of MHC-based approaches: the comparative costs, cost-effectiveness, and return on investment of alternative genotyping platforms and of MHC-informed breeding schemes were considered beyond its scope, which is focused on the structural, evolutionary, and immunological biology of the herbivore MHC. Dedicated economic evaluations, integrating platform-specific genotyping costs with herd- and population-level economic modeling, will therefore be an essential focus of future work before MHC-based typing and selection strategies can be recommended for routine implementation.

Future research should integrate long-read sequencing, pangenomics, multi-omics, single-cell technologies, and artificial intelligence with functional immunological studies to improve our understanding of MHC structure, evolution, and immune function. Equally important will be the establishment of standardized MHC databases, large-scale population resources, and cross-species comparative analyses to facilitate robust genotype–phenotype validation. As experimental evidence continues to accumulate, MHC-informed approaches may provide valuable tools for disease-resistance breeding, vaccine design, animal health management, and the conservation of genetic diversity in herbivore populations. Continued interdisciplinary research will be essential to translate emerging genomic discoveries into reliable applications for livestock production and wildlife conservation.

## Figures and Tables

**Table 1:** Herbivore MHC classification and genetic loci (data source: IPD database).

Species	Scientific Name	MHC Nomenclature	Classical MHC Class I Loci	Non-Classical MHC Class I Loci	Representative MHC Class II Loci
Cattle	*Bos taurus*	BoLA	*BoLA-1*, *BoLA-2*, *BoLA-3*,	*BoLA-NC1*, *BoLA-NC2* *	*BoLA-DQA*, *DQA1*, *DQB*, *DRA*, *DRB1*, *DRB2*, *DRB3*, *DRB4*, *DRB5*
Buffalo	*Bubalus bubalis*	BuLA	Homologous to cattle *BoLA-1*, *BoLA-2*, *BoLA-3*	*BuLA-NC* *	*BuLA-DQA*, *DQB*
Sheep	*Ovis aries*	OLA	*OLA-1* *, *OLA-2* *	*OLA-N*	*OLA-DRA*, *DRB1*, *DQA1*, *DQA2*, *DQB1*, *DQB2*
Goat	*Capra hircus*	CLA	Homologous to sheep OLA	*CLA-N* *	*CLA*- *DRB1*, *DRB3*
Horse	*Equus caballus*	ELA	*ELA-1*, *ELA-2*, *ELA-3*, *ELA-16*	*ELA-N*	*ELA-DMB*, *DOB*, *DQA1*, *DQA2*, *DQA3*, *DQB1*, *DQB2*, *DQB3*, *DRA*, *DRB1*, *DRB2*, *DRB3*

Notes: Data source: IPD-MHC database. BoLA = bovine leukocyte antigen; BuLA = buffalo leukocyte antigen; OLA = ovine leukocyte antigen; CLA = caprine leukocyte antigen; ELA = equine leukocyte antigen; Classical MHC class I genes are highly polymorphic and mainly present endogenous peptides to CD8^+^ T cells; Non-classical MHC class I genes (e.g., *BoLA-NC1*, *OLA-N*, *ELA-N*) generally exhibit limited polymorphism and primarily participate in immune regulation rather than antigen presentation; “Representative loci” indicate the major functional loci reported to date; copy number and gene content may vary among breeds and genome assemblies; * indicates loci that remain incompletely annotated or whose nomenclature is not yet fully standardized.

**Table 2:** Representative summary of conflicting MHC-disease associations in herbivores and their probable explanatory factors.

MHC Locus/Allele	Disease/Phenotype	Conflicting Findings	Probable Causes
*BoLA-DRB3* (Cattle)	Mastitis resistance	DRB3*011:01 protective in European Holsteins; susceptibility-associated in Brazilian crossbreeds	Pathogen species variation (*Staphylococcus* vs. *Streptococcus* spp.); regional microbiome differences; breed background effects
*OLA-DRB1* (Sheep)	Gastrointestinal nematode resistance	Resistance alleles effective in Scottish Blackface; no protective effect confirmed in Merino under same parasite challenge	Breed-specific MHC groove architecture; host genetic background; parasite immune evasion strategies varying by isolate
*ELA-II* (Horse)	Insect bite hypersensitivity	Consistent ELA-II risk haplotypes in Icelandic and Dutch Warmblood horses; not confirmed in Friesians	Geographic variation in Culicoides allergen profiles; population-specific linkage disequilibrium patterns
*BoLA-DRB3* (Cattle/Buffalo)	Foot-and-mouth disease (FMD)	Protective alleles identified for serotype O not protective for serotypes A or Asia-1	Pathogen strain/serotype-specific peptide-MHC binding affinity differences; antigenic variation among FMD strains
*BoLA-DRB3* (Cattle)	BLV proviral load	DRB3*009:02 resistance association confirmed in Japanese Holstein populations; results variable in European and South American herds	Methodological differences (PCR-RFLP vs. high-resolution NGS); population-specific allele frequency distributions; environmental cofactors
Multiple MHC loci (general)	Cross-study comparisons	Older serological typing studies frequently conflict with contemporary NGS-based studies for the same reported associations	Resolution limits of serology vs. sequence-based allele discrimination; inconsistent phenotype definitions across study designs

**Table 3:** Comparative analysis of MHC-mediated disease resistance and susceptibility mechanisms in herbivores.

Disease Category	Pathogen Type	Resistance Mechanism	Susceptibility Mechanism	Immune Outcome (Resistant)	Immune Outcome (Susceptible)	References
Parasitic	Nematode (Sheep)	Efficient peptide binding (OLA-DRB1*1101)	Suboptimal MHC–peptide interaction	Strong CD^4+^ response; low parasite burden	Weak T cell activation; high parasitemia	[89]
Parasitic	Bacterial–fungal (Sheep)	Optimal antigen presentation (OLA-DQA2*1201)	Poor peptide compatibility (*1101)	Enhanced helper T cells; tissue healing	Impaired immune response; chronic lesions	[91]
Bacterial	Mastitis (Cattle)	MHC-II neutrophil recruitment (*BoLA-DRB3*)	Inefficient immune cell localization	Rapid bacterial clearance; resolved infection	Persistent infection; subclinical disease	[93,96]
Viral	FMD (Cattle/Buffalo)	High-affinity peptide presentation (*BoLA-DRB3* HaeIII AA)	Low-affinity MHC binding	Robust humoral & cellular immunity	Weak antibody response; high viremia	[98,99,100]
Viral	EHV-1 (Horse)	Efficient antigen presentation (ELA I, position 173)	Reduced receptor binding capacity	Complete viral clearance; no recurrence	Persistent infection; neurological complications	[105,106,107]
Neoplastic	Equine sarcoidosis (Horse)	Innate immune activation (ELA II, KLRA)	Deficient NK cell signaling	Controlled tumor growth; low recurrence	Uncontrolled proliferation; high recurrence	[97,110,111,112]
Viral	BLV (Cattle)	Proviral suppression (BoLA-DRB3*009:02)	High proviral replication (*015:01)	Reduced vertical transmission; low load	High perinatal transmission; persistent viremia	[103,104]

**Table 4:** Comparison of sequencing platforms for herbivore MHC typing: technical characteristics, advantages, limitations, and recommended research contexts.

Platform	Read Length/Accuracy	Key Advantages	Limitations and Best Use Context
Targeted amplicon NGS (Illumina MiSeq/NextSeq)	2 × 250–300 bp; >99.9% accuracy	High throughput; lowest cost per sample; well-validated pipelines for key loci (e.g., *BoLA-DRB3* exon 2, *OLA-DRB1*)	Cannot resolve full haplotypes; allele dropout in duplicated regions; limited to targeted loci. BEST FOR: large-scale population screening
Whole-genome NGS (Illumina NovaSeq/HiSeq)	2 × 150 bp; >99.9% accuracy	Broad genomic coverage; compatible with multi-purpose WGS datasets; captures novel variants beyond targeted regions	Requires high depth (>100×) for MHC resolution; complex MHC-specific bioinformatics; short reads fail in highly repetitive regions. BEST FOR: multi-purpose datasets; research groups with existing WGS resources
PacBio HiFi (CCS) long-read sequencing	>10–15 kb; >99.9% accuracy	Full MHC haplotype phasing; structural variant detection; resolves paralog ambiguity; enables de novo locus assembly	High cost per sample; stringent HMW DNA quality requirements; lower throughput per run. BEST FOR: haplotype characterization; de novo annotation of novel species; rare/endangered species with complex MHC
Oxford Nanopore (ONT) long-read sequencing	>10–100+ kb; 97–99% accuracy	Flexible throughput; portable FieldSeq potential; real-time base calling; long reads span repetitive regions	Higher per-base error rate than PacBio; requires error correction for accurate allele calling. BEST FOR: field sampling; rapid haplotype screening; exploratory structural variant detection
Capture-based enrichment + NGS	2 × 150 bp (NGS backbone)	Selectively enriches MHC region; compatible with degraded or low-quantity DNA; reduces sequencing depth needed vs. WGS	Panel design requires reference genome; may miss novel structural variants outside capture region. Best for: conservation genetics; wildlife samples; museum specimens

## Data Availability

No new data were created or analyzed in this study. Data sharing is not applicable to this article.
